# Efficacy and Associated Drug Exposures of Isavuconazole and Fluconazole in an Experimental Model of Coccidioidomycosis

**DOI:** 10.1128/AAC.02344-20

**Published:** 2021-05-18

**Authors:** Laura L. Kovanda, Gabriele Sass, Marife Martinez, Karl V. Clemons, Hasan Nazik, Therese M. Kitt, Nathan Wiederhold, William W. Hope, David A. Stevens

**Affiliations:** a Astellas Pharma Global Development, Inc., Northbrook, Illinois, USA; b California Institute for Medical Research, San Jose, California, USA; c University of Texas Health Science Center at San Antonio, San Antonio, Texas, USA; d Antimicrobial Pharmacodynamics and Therapeutics, Department of Molecular and Clinical Pharmacology, Institute of Translational Medicine, University of Liverpool, Liverpool Health Partners, Liverpool, United Kingdom; e Royal Liverpool and Broadgreen University Hospital Trust, Liverpool Health Partners, Liverpool, United Kingdom; f Stanford University School of Medicine, Division of Infectious Diseases and Geographic Medicine, Stanford, California, USA

**Keywords:** coccidioidomycosis, isavuconazole, fluconazole, antifungal therapy, *Coccidioides* spp., endemic mycoses, isavuconazonium sulfate, pharmacodynamics, pharmacology, Valley fever

## Abstract

Coccidioides spp. are important pathogens in regions where they are endemic, and new treatment options are needed. Here, isavuconazonium sulfate (ISAVUSULF) and fluconazole (FLU) were evaluated in experimental disseminated coccidioidomycosis to characterize drug exposures associated with efficacy. Broth macrodilution was performed on Coccidioides isolates to measure minimal effective concentrations (MEC) and minimal fungicidal concentrations (MFC). Mice were inoculated with Coccidioides posadasii (Silveira strain). Treatment started 4 days postinoculation. In model 1, mice were treated for 19 days, followed by 30 days of off-therapy observation, measuring survival through day 49 and residual fungal burden. Treatments included ISAVUSULF (prodrug; 186, 279, or 372 mg/kg twice daily), FLU (20 or 100 mg/kg once daily), and no treatment. Model 2 included 7-day treatment with ISAVUSULF (prodrug; 74.4, 111.6, or 148.8 mg/kg twice daily), FLU (20 or 100 mg/kg once daily), and no treatment. Serial plasma and tissues samples were obtained for pharmacokinetics (PK) and fungal burden measurement, respectively. Fifty percent minimal effective concentration (MEC_50_) values were 0.39 mg/liter (isavuconazole [ISAV]) and 12.5 mg/liter (FLU). Treatment with ISAVUSULF186 or with either FLU dose resulted in higher survival compared to that in the untreated group. Treatment with ISAVUSULF186 or ISAVUSULF279 twice daily or FLU100 reduced fungal burden in all organs (model 1). In model 2, a >1 log_10_ CFU/organ reduction was demonstrated, with ISAV area under the concentration-time curve (AUC) values achieved with 111.6 mg/kg twice daily (56.8 mg · h/liter) in the spleen and liver. FLU AUC values of 100 and 500 mg·h/liter for 20 and 100 mg/kg doses, respectively, resulted in a >1 log_10_ CFU/organ mean reduction in all organs. ISAVUSULF and FLU improved survival and reduced fungal burden. Increasing plasma drug exposures resulted in decreases in fungal burden.

## TEXT

Coccidioidomycosis, often called “Valley fever,” is a fungal infection caused by the endemic dimorphic fungi Coccidioides immitis and Coccidioides posadasii, which are typically found in the soil in the western portion of the United States, areas of Mexico, and parts of Central and South America (1). This infectious disease represents an important unmet need, and further information is needed to better guide treatment paradigms. Although there is significant disease burden reported yearly in regions where Coccidioides spp. are endemic, clinical studies of new agents that demonstrate *in vitro* activity against this genus are sparse or have not been conducted. Therefore, treatment is often guided by history, experimental data, and case reports.

Approximately 150,000 cases are reported annually in the United States, with only about a third of patients seeking medical attention, most often those patients with pneumonia (2). The spectrum of disease ranges from community-acquired pneumonia to more severe hematogenous dissemination (3). Infections are contracted by inhaling arthroconidia, which change morphologically in the host into spherules in tissues. The spherules contain endospores and, if they rupture, spread the infection via hematogenous or lymphatic dissemination (4). Therapeutic options are limited to oral triazole antifungals, such as fluconazole or itraconazole, and, in more severe infections, intravenous or intrathecal amphotericin B (2). Duration of treatment can range from months to years, depending on the evidence of residual disease, symptoms, or presence of disseminated disease. Disease severity can differ based on differences in immune responses to the infection (5).

Isavuconazole (ISAV), the active moiety of the triazole antifungal prodrug isavuconazonium sulfate (ISAVUSULF), has demonstrated potent *in vitro* activity against isolates of *Coccidioides* spp. Clinical studies in small cohorts of patients have reported good clinical outcomes with this triazole (6, 7). Experimental models with *Coccidioides* spp. and ISAVUSULF have not been performed to date. In addition, there is a paucity of data in experimental models demonstrating the exposure-response relationship of existing antifungal agents for coccidioidomycosis. ISAV and fluconazole (FLU) have been extensively studied in other infection models where pharmacokinetic-pharmacodynamic (PK-PD) relationships have been established (8–16).

We sought to characterize the *in vivo* efficacy of ISAVUSULF in an established model of coccidioidomycosis using FLU as an active control, followed by a study to characterize the PK-PD relationship between ISAV and FLU exposures and outcome in a mouse model of experimental coccidioidomycosis.

## RESULTS

### *In vitro* susceptibility and fungicidal testing.

The minimal effective concentrations (MEC) and minimal fungicidal concentration (MFC) of 32 strains of *Coccidioides* spp. not definitively identified to the species level, but presumed to be C. immitis, based on geographic location of the patient, except for one strain of C. posadasii (isolate 1), are shown in Table 1. The MEC values (2-fold dilution range tested from 0.39 mg/liter to 100 mg/liter) were ≤0.39 to 1.56 mg/liter for ISAV (geometric mean, 0.495 mg/liter), and 6.25 to >100 mg/liter (geometric mean, 10.98 mg/liter) for FLU. Fifty percent minimal effective concentration (MEC_50_) and MEC_90_ values were, respectively, 0.39 and 0.78 mg/liter for ISAV and 12.5 and 50 mg/liter for FLU. ISAV and FLU MFC values trended higher than the MEC values, suggesting fungistatic activity against *Coccidioides* spp. for both drugs *in vitro*. For ISAV, the isolates were inhibited only to “trace” values below the MEC, suggesting “trailing” in the tubes, except for isolate 4, which showed a clear tube even at 0.39 mg/liter. The trailing phenomenon occurred for some isolates for FLU. Subculturing of the trace growth seen with both drugs did result in few (4 to 25) colonies of viable *Coccidioides* in each instance. The ISAV and FLU MEC of the *Coccidioides posadasii* strain Silveira used for the *in vivo* experiments was ≤0.39 and 3.13 mg/liter, respectively.

**TABLE 1:** Summary of MEC and MFC values for isavuconazole and fluconazole^*a*^

Statistic	Isavuconazole		Fluconazole	
	MEC (mg/liter)	MFC (mg/liter)	MEC (mg/liter)	MFC (mg/liter)
*N*	32	18	32	18
MEC_50_	0.39	—	12.5	—
MEC_90_	0.78	—	50	—
Geometric mean	0.495	2.781	10.98	38.19
Geometric SD	1.517	6.142	2.979	2.367
Range	≤0.39 to 1.56	≤0.39 to 100	0.78 to >100	12.5 to >100

a MEC, minimal effective concentration; MFC, minimal fungicidal concentration; —, not done.

### Animal models of coccidioidomycosis.

**FIG 1:**
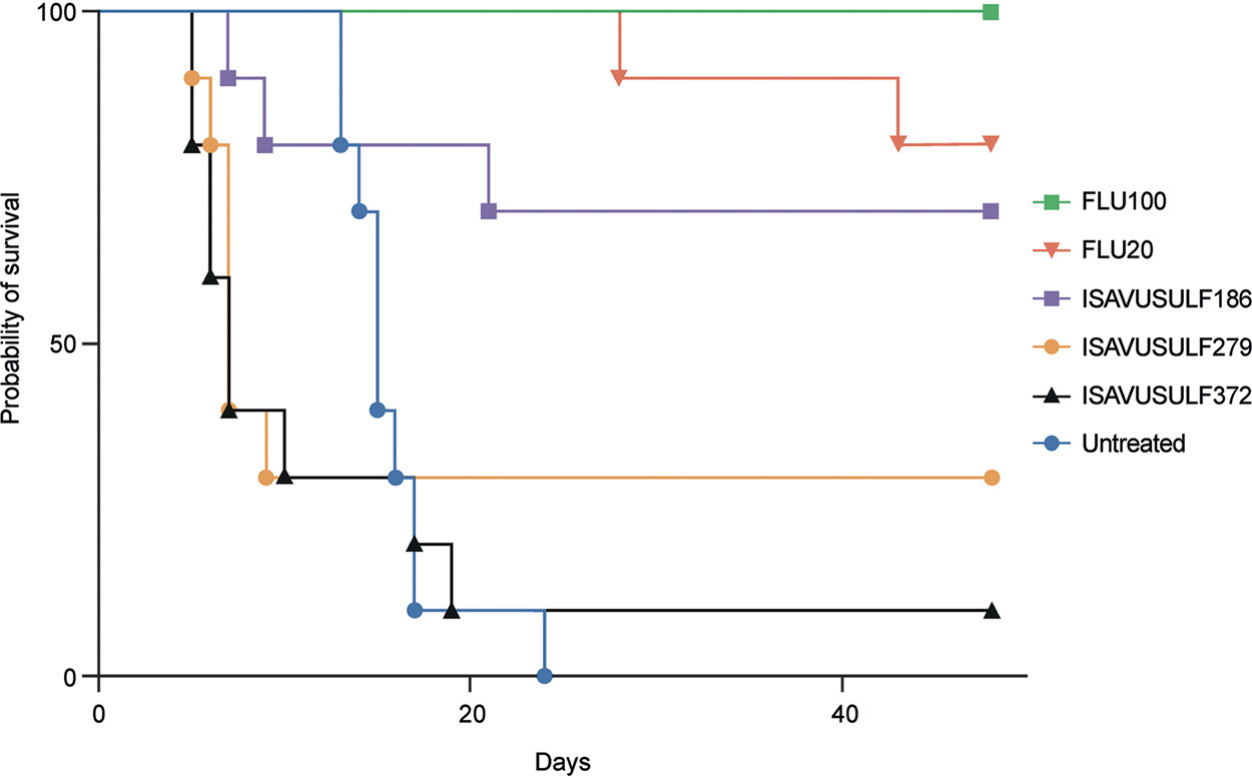
Model 1. Survival after treatment duration of 19 days and 30 days off therapy. FLU100, fluconazole 100 mg/kg; FLU20, fluconazole 20 mg/kg; ISAVUSULF186, isavuconazonium sulfate 186 mg/kg twice daily; ISAVUSULF279, isavuconazonium sulfate 279 mg/kg twice daily; ISAVUSULF372, isavuconazonium sulfate 372 mg/kg twice daily.

**(i) Model 1: prolonged therapy.** *(a) Survival.* Treatment with FLU 20 mg/kg and 100 mg/kg resulted in 80% and 100% survival through day 49, respectively, significantly higher than that of controls (all *P* values = 0.0001; log-rank test) (Fig. 1). The initial doses tested for ISAVUSULF resulted in toxicity with survival rates of 70%, 30%, and 10% for the 186 mg/kg, 279 mg/kg, and 372 mg/kg twice daily dose groups (equivalent to 100 mg/kg, 150 mg/kg, and 200 mg/kg ISAV twice daily), respectively (Fig. 1). Survival in the lowest ISAVSULF dose group was significantly higher than that in untreated controls (*P* = 0.0029; log-rank test). ISAVUSULF doses were subsequently lowered for model 2.

**FIG 2:**
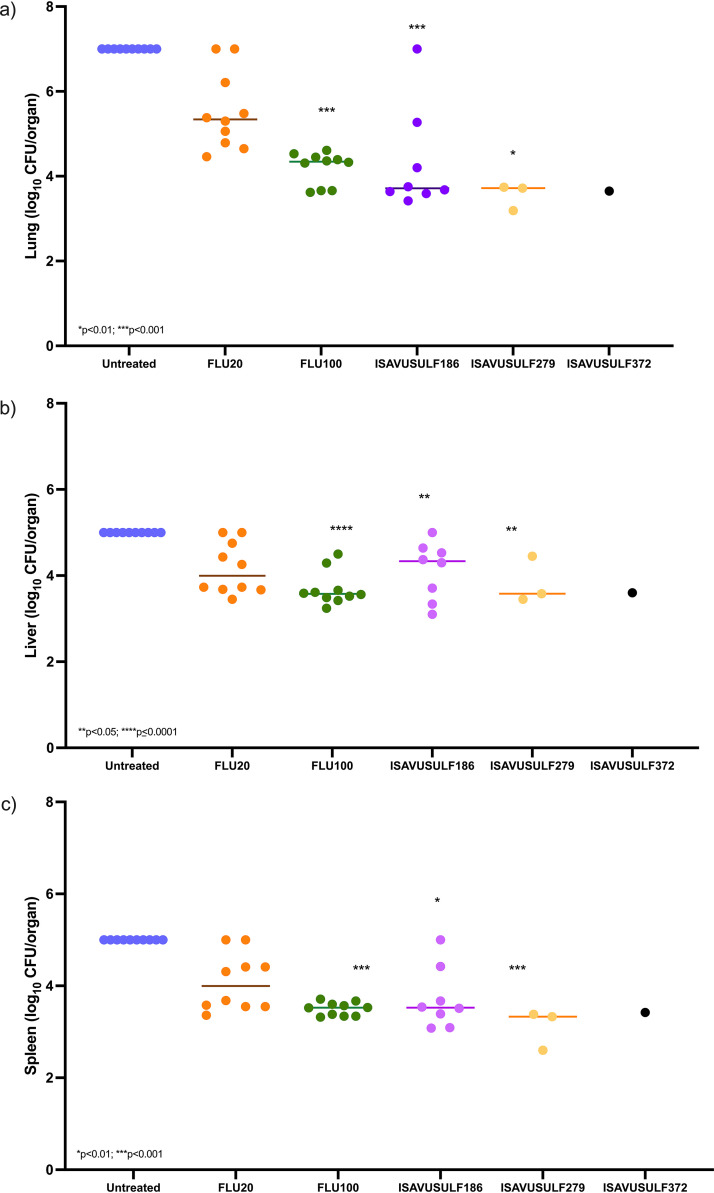
Model 1. Fungal burden by treatment groups in the lungs (a), liver (b), and spleen (c) after 19 days on therapy and 30 days without. FLU100, fluconazole 100 mg/kg; FLU20, fluconazole 20 mg/kg; ISAVUSULF186, isavuconazonium sulfate 186 mg/kg twice daily; ISAVUSULF279, isavuconazonium sulfate 279 mg/kg twice daily; ISAVUSULF372, isavuconazonium sulfate 372 mg/kg twice daily. Individual values; bar represents median. *P* values are for comparisons between each dose group and the untreated group.

*(b) Fungal burden.* ISAVUSULF at doses of 186 mg/kg and 279 mg/kg twice daily and FLU 100 mg/kg once daily significantly reduced fungal burden in the lungs, spleen, and liver compared to that in controls (*P* values of <0.001 [lung], <0.05 [liver], <0.01 [spleen], and <0.01 [lung], <0.05 [liver], <0.001 [spleen] for ISAVUSULF 186 mg/kg and 279 mg/kg twice daily, respectively; *P* values of <0.001 [lung and spleen] and ≤0.0001 [liver] for FLU 100 mg/kg doses; Kruskal-Wallis test, *P* values adjusted for multiple comparisons using Dunn’s multiple-comparison test) (Fig. 2a to c). FLU 20 mg/kg significantly reduced fungal burden compared to that of controls in the liver only (*P* < 0.05; Kruskal-Wallis test). In the lungs, FLU showed a dose-dependent reduction in log_10_ CFU/g. Due to the toxicity at the high ISAVUSULF dose (372 mg/kg twice daily), CFU comparisons were not possible for this group.

**(ii) Toxicity study.** Uninfected mice receiving lower doses of ISAVUSULF of 74.4 mg/kg, 111.6 mg/kg, and 148.8 mg/kg twice daily (equivalent to 40 mg/kg, 60 mg/kg, and 80 mg/kg ISAV twice daily) for 19 days showed no signs of distress, such as ruffled fur, lethargy, or severe agitation. There were no significant differences in mouse weight over time for each of the dose groups, and all mice survived the duration of the study (through day 49) (data not shown). Therefore, these doses were chosen for the next group of experiments.

**FIG 3:**
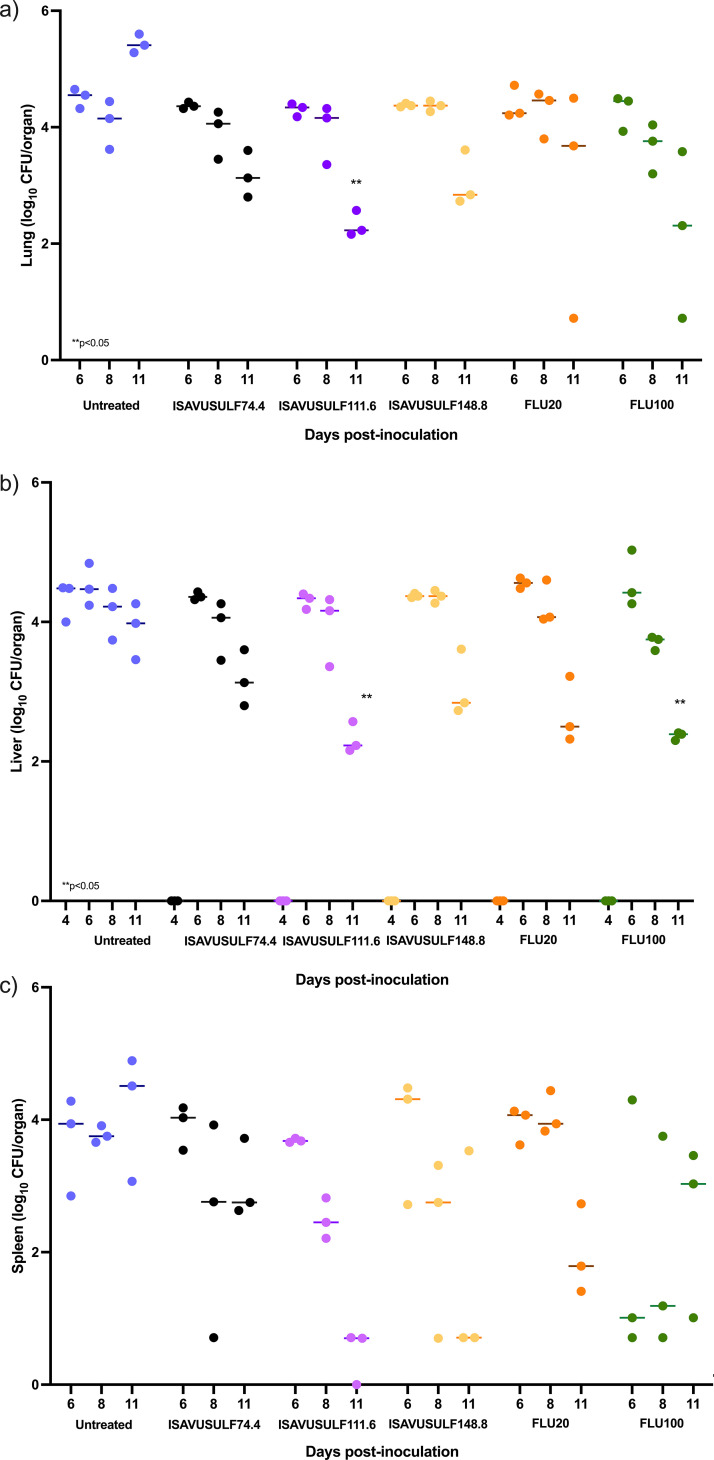
Model 2. Change in fungal burden over time by treatment group in the lungs (a), liver (b), and spleen (c) after 7 days of therapy. FLU100, fluconazole 100 mg/kg; FLU20, fluconazole 20 mg/kg; ISAVUSULF74.4, isavuconazonium sulfate 74.4 mg/kg twice daily; ISAVUSULF111.6, isavuconazonium sulfate 111.6 mg/kg twice daily; ISAVUSULF148.8, isavuconazonium sulfate 148.8 mg/kg twice daily. Individual values; bar represents median. *P* values are for comparisons between each dose group and the untreated group.

**(iii) Model 2: PK-PD.** *(a) Fungal burden in lungs.* Infection in the lungs was not detected until day 6 in all groups (Fig. 3a). Significant increases in fungal burden over time by day 11 were seen in the lungs of the untreated group (*P* ≤ 0.01; Mann-Whitney U test). Treatment with oral ISAVUSULF at doses of 111.6 mg/kg twice daily resulted in significant reductions in lung fungal burden by day 11 (*P* < 0.05; Kruskal-Wallis test, adjusted using Dunn’s multiple-comparison test).

*(b) Fungal burden in the liver.* Fungal burden in the liver was >4 log_10_ CFU/organ of tissue on day 4 postinoculation in the untreated group and remained relatively consistent over the study duration (Fig. 3b). Significant reductions in the liver log_10_ CFU/organ versus that of controls were seen by day 11 for the 111.6 mg/kg twice daily ISAVUSULF group and the FLU at 100 mg group (*P* < 0.05; Kruskal-Wallis test, adjusted using Dunn’s multiple-comparison test).

*(c) Fungal burden in the spleen.* Fungal infection was not detected in the spleen until day 6 postinoculation and remained constant over time in the untreated group after detection (no significant increase after day 6) (Fig. 3c). Reductions in log_10_ CFU/organ compared to that of controls were not significant for any dose group by day 11 (*P* > 0.05; Kruskal-Wallis test, adjusted using Dunn’s multiple-comparison test).

### Exposure-response relationship.

**FIG 4:**
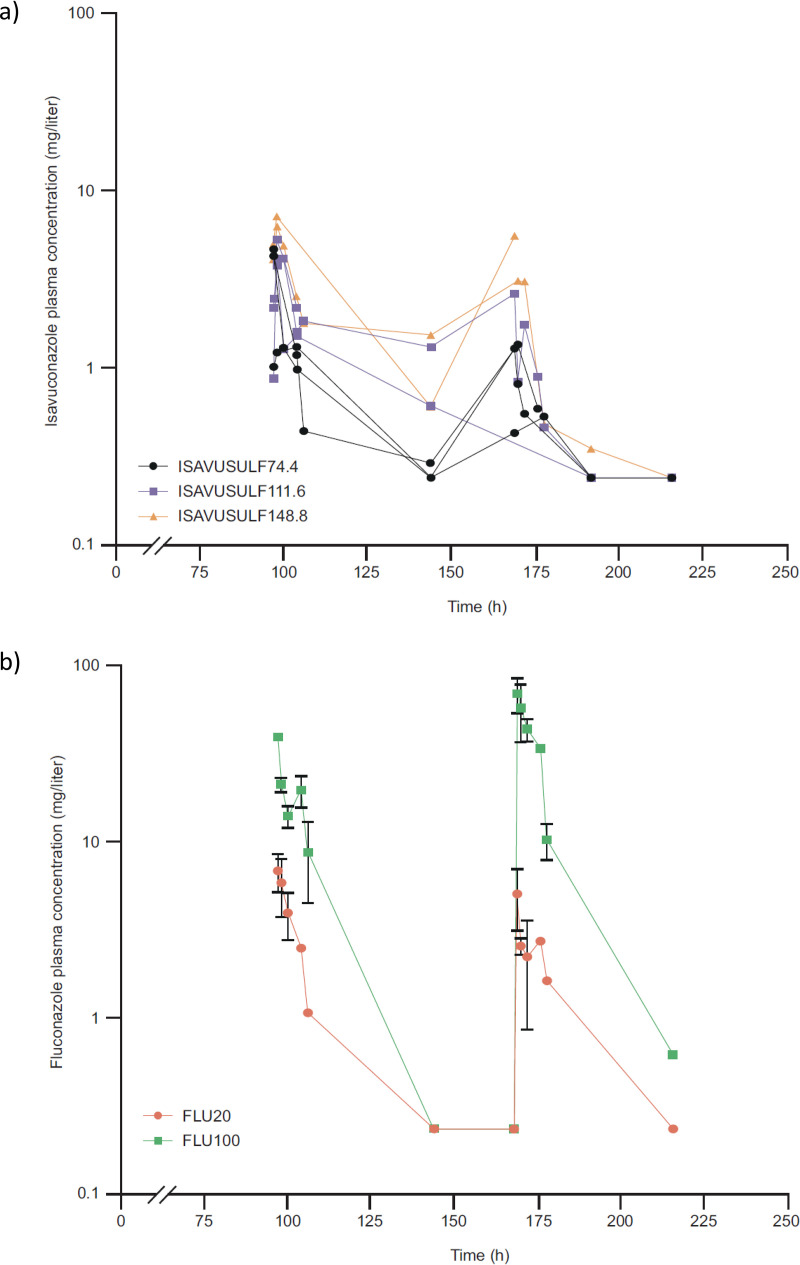
Model 2. Isavuconazole (a) and fluconazole (b) plasma concentrations over time as collected on days 4 and 10 (i.e., treatment days 1 and 7) of the experiment. FLU100, fluconazole 100 mg/kg; FLU20, fluconazole 20 mg/kg; h, hour; ISAVUSULF74.4, isavuconazonium sulfate 74.4 mg/kg twice daily; ISAVUSULF111.6, isavuconazonium sulfate 111.6 mg/kg twice daily; ISAVUSULF148.8, isavuconazonium sulfate 148.8 mg/kg twice daily.

**FIG 5:**
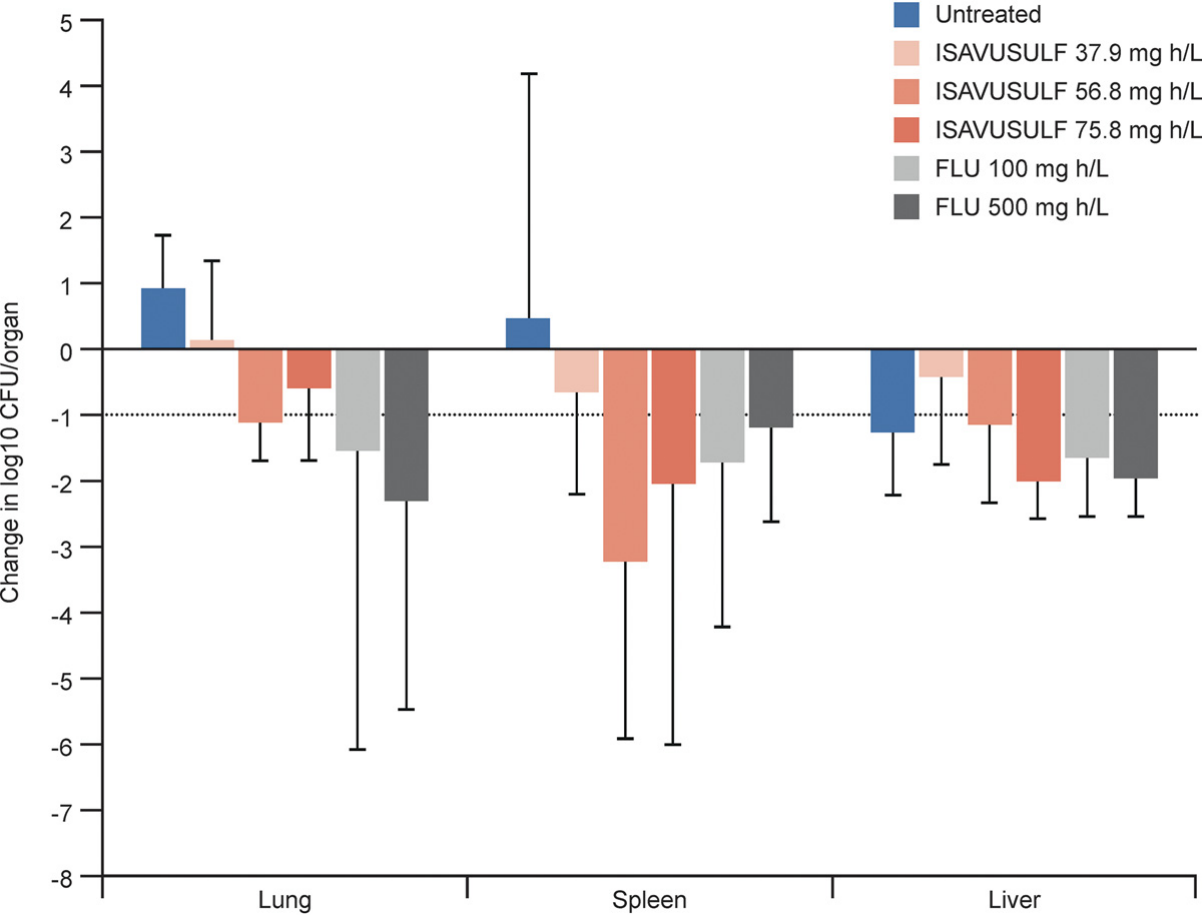
Model 2. Mean change in fungal burden at day 11 by area under the concentration-time curve (AUC). FLU, fluconazole; ISAVUSULF, isavuconazonium sulfate. Horizontal dashed line at 1 log reduction. Bars represent mean change and variance shown as 95% confidence intervals. ISAVUSULF doses given twice daily; FLU given once daily.

**(i) Isavuconazole.** The ISAV plasma concentrations over time are shown in Fig. 4a. Estimated exposures (area under the concentration-time curve [AUC] values) for each oral ISAVUSULF total daily dose (11) were 37.9 mg · h/liter, 56.8 mg · h/liter, and 75.8 mg · h/liter (80 mg/kg, 120 mg/kg, and 160 mg/kg total daily ISAV-equivalent dose, respectively [model 2 dosages]). Drug exposures of 56.8 mg · h/liter or greater or an AUC:MIC ratio of 454.4 (ISAV MIC of 0.125 mg/liter for *C. posadasii* strain Silveira [ATCC 28-868] tested at 80% inhibitory level; data on file at the University of Texas Fungus Testing Laboratory) were necessary to demonstrate a decline in fungal burden of >1 log_10_ CFU/organ in the spleen and liver. A less than 1 log_10_ CFU/organ mean change (−0.593; standard deviation [SD], ±0.434) was demonstrated in the lungs at these exposures (Fig. 5).

**(ii) Fluconazole.** The FLU plasma concentrations over time are shown in Fig. 4b with AUC values of 100 mg · h/liter and 500 mg · h/liter for the 20 mg/kg and 100 mg/kg doses, respectively (14) or an AUC:MIC ratio of at least 12.5 (FLU MIC for *C. posadasii* strain Silveira [ATCC 28-868] of 8 mg/liter for *C. posadasii* tested at 80% inhibitory level; data on file at University of Texas Fungus Testing Laboratory). At these exposures, a >1 log_10_ CFU/organ mean decline in fungal burden was achieved in all organs (Fig. 5).

## DISCUSSION

*Coccidioides* spp. causes significant disease burden across regions where they are endemic in North and South America. The triazole antifungal agents FLU and itraconazole are commonly used to treat these infections, and there are limited data with newer agents, such voriconazole and posaconazole (1). ISAVUSULF has potent *in vitro* activity and limited available clinical efficacy data against these endemic fungi, but has not been previously studied in experimental coccidioidomycosis models (6, 7). In addition, exposure-response relationships for antifungal agents in the setting of coccidioidomycosis have not been extensively characterized in experimental models. The results shown here describe the efficacy of ISAVUSULF and FLU against experimental coccidioidomycosis. In addition, we studied a PK-PD murine model to associate exposures with respective changes in fungal burden.

In model 1 (prolonged therapy model), initial doses of ISAVUSULF were not tolerated by the mice, which led to the need for a toxicity study to find the most appropriate dosages for further experiments. ISAVUSULF has been used extensively in mice in a broad range of infection types and dosage regimens (9–11, 17, 18). Reports of toxicity in mice have been made previously, but not consistently across mouse models, and the occurrence is dose dependent (17). The model 1-calculated ISAV AUC values at the total daily doses used ranged from 95 to 189 mg · h/liter, twice the human exposure at the clinical dosage regimen. In addition to decreasing the dosages for model 2, we modified the procedure for drug preparation as described previously (19). The change to the procedure was to adjust the pH of USP water to 3.99 to 4.01 daily, filter sterilize it, add the filtrate to the aliquots of ISAVUSULF, and vortex the mix gently while mixing. With this modified procedure, no additional toxicity was observed in the animals. The mice tolerated the new dosage regimens, which allowed the PK-PD portion of the study to proceed, albeit at lower ISAVUSULF doses, which achieved drug exposures below those achieved in adults after administration of the clinical recommended dosage (20, 21). Despite the lower exposures, the mid and high doses of ISAVUSULF performed on par with FLU. It is of note that the FLU concentrations and calculated AUC values were much higher than ISAV AUC values at the dosages administered in model 2 (Fig. 4a and b).

The PK-PD model studied here afforded an opportunity to explore the exposure associated with efficacy for ISAVUSULF and FLU in the treatment of coccidioidomycosis. We showed that reduction in fungal burden over time was associated more often with higher exposures in the lung and liver. The burden data in the spleen were relatively flat, and the fungal burden overall was not as impressive in this organ as in the other two. There was considerable variation in the measured fungal burden at each time point tested in all organs, especially in the spleen; however, this was somewhat expected given the short duration of treatment (10 days).

FLU has been well established as a recommended treatment regimen in coccidioidomycosis (2); however, data for ISAVUSULF for treatment of this disease are sparse. *In vitro* data from this study suggest that both of these azoles have fungistatic activity against *Coccidioides* spp. A small series of cases treated with ISAVUSULF from a clinical trial have been reported (6). In that study, nine subjects with pulmonary infections caused by *Coccidioides* spp. were treated, and treatment durations ranged from 180 to 183 days (the maximum allowable duration in this study was 180 days). Successful therapy as defined by the study (combined clinical, mycological, and radiological response at the end of therapy [EOT] as adjudicated by an independent review committee) was achieved in 5 of 9 patients. The remaining subjects were classified as stable disease at the EOT because of remaining residual radiographic abnormalities. Mean drug exposures (AUC_avg_) in these patients was 94.0 mg · h/liter (20), which is much higher than calculated ISAV AUC values in mice from model 2 at all ISAVUSULF dosages tested. Figure 5 shows us that increasing drug exposures resulted in significant changes in fungal burden in the liver for ISAVUSULF and FLU. In the lung and spleen, these findings were less pronounced for ISAVUSULF than for FLU, but nonetheless, ISAVUSULF AUC values at or above 56.8 mg · h/liter or an AUC:MIC ratio of 454.4 was sufficient to reduce fungal burden over time for all organs tested, including >1 log_10_ CFU/organ reduction in the liver and spleen over this short treatment duration.

Understanding the PK-PD relationships to support treatment decisions for patients is important. Drug exposures (AUC values) of ISAV after administration of the clinical dosage regimen are approximately 94 mg · h/liter (20), and at this AUC, patients should be well above the estimated PD requirement with the recommended clinical dosage regimen for organisms with an MIC value up to approximately 0.125 mg/liter. For FLU, dosages of 400 to 1,200 mg/day are recommended by the Infectious Diseases Society of America (IDSA) guidelines for coccidioidomycosis (2). At these doses and by our calculations, total drug exposures should be enough to treat organisms with MICs of up to approximately 32 or 64 mg/liter, respectively. These estimates of course are general and do not take into account specific sites of infection, such as CNS. In addition, the below limitations should be considered when contemplating these suggested associations.

There are several limitations of these experiments. First, the dosage of ISAVUSULF estimated to be necessary prior to the experiments was thought to be higher than those tested in model 2. It is anticipated that higher dosages/exposures would have provided an even clearer dose-dependent relationship for ISAVUSULF; however, this was not possible due to toxicity in the initial experiments. Second, it has been established that coccidioidomycosis requires long durations of therapy and established experimental mouse models test potential therapies for longer than our PK-PD model for that reason. It is possible that had the PK-PD experiments run for more than 10 days (>7 days on treatment), we might have seen more drastic declines in fungal burden. However, given the intensive sampling required for a PK-PD study such as this, we proceeded with a shorter duration of therapy. In addition, the isolate tested was selected for this study owing to its predictable behavior in challenge models. However, we tested only one isolate with one susceptibility profile, which limited the ability to extend the findings from this study against isolates with reduced *in vitro* susceptibility to ISAV. This would have also allowed us to run a more extensive PK-PD mathematically linked model to characterize the exposure-response relationship in more detail.

In this study, ISAVUSULF and FLU caused a significant reduction in fungal burden in mice compared to that of controls, in a dose-dependent manner, after prolonged therapy with a follow-up period. In addition, model 2 represents one of the first attempts to associate drug exposures with the treatment of experimental coccidioidomycosis. Increasing exposures resulted in decreases in fungal burden over time. These findings should be tested further but demonstrate the usefulness of both therapies in the setting of coccidioidomycosis and emphasize the importance of dose in successful treatment.

## MATERIALS AND METHODS

### *In vitro* susceptibility and fungicidal testing.

Thirty-two strains of *Coccidioides* spp. were tested in a biosafety level 3 facility. Broth macrodilution was performed in tubes with an initial inoculum of 10^3^ arthroconidia/ml for both ISAV and FLU, with a 2-fold dilution range for both from 0.39 to 100 mg/liter, in RPMI 1640 medium incubated at approximately human body temperature (35 to 37°C) on a slow rotating shaker, as previously described (22–24). The MEC value, the lowest concentration of an antimicrobial agent that leads to the growth of small, rounded, compact hyphal forms compared to the hyphal growth seen in the growth control well, was determined when the control growth was 4+, and the endpoint was defined as the first tube with only trace growth. MFC was determined by subculturing the isolate to agar and was defined as killing ≥99% of the initial inoculum (25). Both readings occurred after 7 days of incubation. Quality control was performed each day against Candida kefyr strain SA with acceptable criteria of ≤0.39 mg/liter for both ISAV and FLU.

### Organism for *in vivo* models.

The well-characterized Silveira strain of *Coccidioides posadasii* (ATCC 28-868; isolate 1) was grown on glucose-yeast extract agar. All handling and testing procedures of the organism were conducted per biosafety level 3 requirements. Housing and accommodations were as per the protocols of the California Institute for Medical Research Animal Facility. Approval of all procedures was obtained from the Institutional Animal Care and Use Committee at the California Institute for Medical Research, San Jose, CA.

### Animal models of coccidioidomycosis (models 1 and 2).

Seven-week-old female CD-1 mice, 10 mice per group, were housed 5 or 6 per cage and provided with sterilized food and acidified water *ad libitum*.

**(i) Model 1: prolonged therapy model.** Mice were challenged intravenously with 310 viable arthroconidia (the morphological form that causes human infection). Treatment initiation occurred 4 days after inoculation to allow infection to become established and continued for 19 days. Mice were monitored for death through 49 days postinfection. Mice surviving through 49 days were euthanized by CO_2_ asphyxiation. The objective of the posttherapy observation period was to compare the fungistatic (therapy prolongs survival only when given drug) versus fungicidal (survival is maintained after drug is stopped) effect *in vivo*.

*(a) Quantification of residual burden in the lungs, liver, and spleen.* Each organ was aseptically removed postmortem and homogenized in 5 ml of sterile saline containing antibacterials. Serial dilutions of the organ homogenates were placed onto agar plates containing cycloheximide and chloramphenicol and incubated at 37°C to determine the number of viable CFU in each organ. For statistical comparisons of fungal burden, CFU for untreated and treated mice that died before day 49 were arbitrarily assigned a CFU value higher than that of the surviving animals (log_10_ 7 CFU/organ) (26, 27).

*(b) Treatments.* Treatment groups included mice receiving ISAVUSULF at doses of 186 mg/kg, 279 mg/kg, or 372 mg/kg twice daily (equivalent to 100 mg/kg, 150 mg/kg, and 200 mg/kg twice-daily of ISAV, respectively) via oral gavage. FLU dose groups included 20 mg/kg or 100 mg/kg once per day. The untreated group received no active treatment.

**(ii) Toxicity study.** Mortality at the doses in model 1 led to a toxicity study at lower doses to determine the best doses to use in model 2. Six-week-old CD-1 female mice were used. Mice were uninfected, and ISAVUSULF was prepared in sterile water and buffered to a pH of 4.0. Treatment groups included mice receiving ISAVUSULF at doses of 74.4 mg/kg, 111.6 mg/kg, and 148.8 mg/kg twice daily (equivalent to 40 mg/kg, 60 mg/kg, or 80 mg/kg of ISAV twice daily). Treatment was for 19 days, and the mice were followed until day 49 after first treatment. Mice were weighed before, during, and after the treatment period. Mice were euthanized by CO_2_ asphyxiation on day 49.

**(iii) Model 2: PK-PD model.** Six-week-old female CD-1 mice were intravenously inoculated with 190 viable arthroconidia. Treatment started 4 days postinoculation and continued for 7 days. Treatment groups included mice receiving oral ISAVUSULF at doses of 74.4 mg/kg, 111.6 mg/kg, and 148.8 mg/kg twice daily; FLU at doses of 20 mg/kg or 100 mg/kg once daily; and untreated controls. On day 11 postinoculation, all surviving mice were euthanized by CO_2_ asphyxiation. Residual burden in the lungs, brain, and spleen was measured on days 4, 6, 8, and 11 postinoculation (three mice per time point per dose group), and organs were processed as described for model 1.

*(a) Pharmacokinetic blood collections.* Sample collection was performed on days 4 and 10 (three per time point per ISAVUSULF or FLU dose group) postinfection at the following time points: 0 (prior to first dose), 1, 2, 8, 12, and 24 h postdosing. Blood samples were taken in EDTA plasma separator tubes at the indicated time points postdrug administration. Plasma was isolated by centrifugation and stored at −80°C prior to testing.

*(b) Bioanalytical assay.* ISAV and FLU levels were measured using a validated ultraperformance liquid chromatography–single-quadrupole mass spectrometry analytical assay (Waters, Inc., Milford, MA) (17). A standard curve was prepared by spiking blank plasma with ISAV (BAL4815; Astellas Pharma Global Development, Inc., Northbrook, IL) or FLU (Sigma, St. Louis, MO), followed by the addition of an internal standard to each sample (valethamate bromide; Sigma). All samples were then buffered and loaded onto conditioned solid-phase extraction (SPE) columns; this was followed by independent washings with 5.0% NH_4_OH and 15.0% methanolic water. The samples were then eluted with 1.0 ml of 100% methanol and 1.0 ml of an acidic methanolic mixture (2% formic acid in methanol). The combined eluates were dried under a stream of nitrogen, and the dried residues were reconstituted with 60:40 acetonitrile/water, injected, and analyzed under specified conditions using a mass to charge ratio (*m/z*) of 438.2 for ISAV and 307.1 for FLU. The lowest limit of quantitation was 0.25 μg/ml for both ISAV and FLU.

**(iv) Statistical analyses. **Model 1 group differences were conducted using a log-rank test for cumulative survival. The Kruskal-Wallis test was used for analysis of fungal burden and adjusted for multiple comparisons using Dunn’s multiple-comparison test for models 1 and 2. The Mann-Whitney U test was utilized for single comparisons. Statistical comparisons were performed in Prism version 8.3.4 (GraphPad Software, San Diego, CA).
